# Trials and Tribulations: Palliative Care Trial Recruitment Approaches and Challenges

**DOI:** 10.1093/geroni/igab046.634

**Published:** 2021-12-17

**Authors:** Susan Enguidanos, Anna Rahman, Sindy Lomeli

**Affiliations:** University of Southern California, Los Angeles, California, United States

## Abstract

In 2017, we received funding form the Patient-Centered Outcomes Research Institute to conduct a large, state-wide, randomized controlled trial to test the effectiveness of a home-based palliative care (HBPC) program within accountable care organizations. Participants were randomized to either HBPC or enhanced usual care, where physicians were provided added training and support in core palliative care practices. Originally, we planned to obtain patient referrals to the trial from primary care physicians, however we were unable to engage primary care physicians in patient identification processes. In this session we will describe the numerous trial modifications made to our trial recruitment methods and the success of each approach. Ultimately, after 20 months of trial recruitment, we had recruited just 28 patients and 10 of their caregivers. Findings from this terminated trial may inform other researchers in development of participant recruitment methods.

